# Supplementary material for: Why we publish where we do: Faculty publishing values and their relationship to review, promotion and tenure expectations
Source: PLoS One. 2020 Mar 11;15(3):e0228914. doi: 10.1371/journal.pone.0228914 (PMC7065820; doi:10.1371/journal.pone.0228914)
Supplement: S1 Table — ANOVA were used for statistical significance tests. (DOCX) [file pone.0228914.s001.docx]

## **Supplementary Materials**

| S1 Table. Mean responses, standard error (SE) and p values for publishing decisions and productivity by tenure status. ANOVA were used for statistical significance tests. | | | | | |
| --- | --- | --- | --- | --- | --- |
| **Variable** | **Tenured** | **SE** | **Non-tenure** | **SE** | **p=** |
| pubs published | 3.15 | 0.92 | 3.24 | 0.91 | 0.467 |
| merit pay | 1.91 | 1.43 | 2.04 | 1.55 | 0.597 |
| readership | 5.03 | 1.27 | 5.02 | 1.23 | 0.989 |
| Journal IF | 4.18 | 1.41 | 4.61 | 1.58 | 0.029 |
| society journal | 3.47 | 1.66 | 3.45 | 1.73 | 0.958 |
| journal read | 4.49 | 1.30 | 4.50 | 1.41 | 0.927 |
| journal peers read | 4.66 | 1.36 | 4.76 | 1.44 | 0.599 |
| journal citations | 3.80 | 1.49 | 4.11 | 1.45 | 0.126 |
| journal prestige | 4.70 | 1.17 | 4.95 | 1.23 | 0.123 |
| open access | 3.23 | 1.62 | 3.50 | 1.51 | 0.216 |
| journal cost | 3.70 | 1.80 | 3.71 | 1.82 | 0.976 |
