# Supplementary material for: Why we publish where we do: Faculty publishing values and their relationship to review, promotion and tenure expectations
Source: PLoS One. 2020 Mar 11;15(3):e0228914. doi: 10.1371/journal.pone.0228914 (PMC7065820; doi:10.1371/journal.pone.0228914)
Supplement: S2 Table — ANOVA were used for statistical significance tests for all variables except for “pubs published”, which is a categorical variable where chi2 tests were used. (DOCX) [file pone.0228914.s002.docx]

| S2 Table. Mean responses, standard error (SE) and p values for publishing decisions and productivity by gender and institution type. ANOVA were used for statistical significance tests for all variables except for “pubs published”, which is a categorical variable where chi2 tests were used. | | | | | | | | | | |
| --- | --- | --- | --- | --- | --- | --- | --- | --- | --- | --- |
| **Variable** | **Female** | **SE** | **Male** | **SE** | **p=** | **R Type** | **SE** | **M Type** | **SE** | **p=** |
| pubs published | 3.05 | 0.88 | 3.28 | 0.96 | 0.084 | 3.30 | 0.89 | 2.89 | 0.93 | 0.000 |
| merit pay | 2.08 | 1.66 | 1.87 | 1.29 | 0.308 | 2.03 | 1.50 | 1.77 | 1.36 | 0.256 |
| readership | 5.00 | 1.25 | 5.02 | 1.30 | 0.927 | 5.11 | 1.16 | 4.82 | 1.47 | 0.073 |
| Journal IF | 4.33 | 1.45 | 4.26 | 1.49 | 0.721 | 4.38 | 1.42 | 4.13 | 1.57 | 0.199 |
| society journal | 3.54 | 1.77 | 3.41 | 1.58 | 0.543 | 3.51 | 1.61 | 3.39 | 1.82 | 0.596 |
| journal read | 4.59 | 1.31 | 4.38 | 1.38 | 0.197 | 4.57 | 1.26 | 4.29 | 1.50 | 0.109 |
| journal peers read | 4.69 | 1.45 | 4.66 | 1.34 | 0.853 | 4.73 | 1.37 | 4.54 | 1.43 | 0.308 |
| journal citations | 3.94 | 1.48 | 3.84 | 1.48 | 0.595 | 3.94 | 1.51 | 3.74 | 1.45 | 0.310 |
| journal prestige | 4.73 | 1.25 | 4.76 | 1.15 | 0.812 | 4.79 | 1.24 | 4.65 | 1.10 | 0.363 |
| open access | 3.24 | 1.59 | 3.38 | 1.60 | 0.488 | 3.24 | 1.55 | 3.43 | 1.69 | 0.386 |
| journal cost | 4.14 | 1.86 | 3.36 | 3.36 | 0.001 | 3.80 | 1.78 | 3.51 | 1.83 | 0.232 |
