# Supplementary material for: Why we publish where we do: Faculty publishing values and their relationship to review, promotion and tenure expectations
Source: PLoS One. 2020 Mar 11;15(3):e0228914. doi: 10.1371/journal.pone.0228914 (PMC7065820; doi:10.1371/journal.pone.0228914)
Supplement: S3 Table — (DOCX) [file pone.0228914.s003.docx]

| S3 Table. Spearman's correlations and p values for publishing decisions and productivity by age | | |
| --- | --- | --- |
| **Variable** | **Age** | **p =** |
| pubs published | -0.109 | 0.064 |
| merit pay | 0.080 | 0.256 |
| readership | -0.026 | 0.662 |
| Journal IF | -0.156 | 0.009 |
| society journal | 0.124 | 0.039 |
| journal read | -0.024 | 0.690 |
| journal peers read | -0.089 | 0.140 |
| journal citations | -0.182 | 0.002 |
| journal prestige | -0.165 | 0.005 |
| open access | 0.097 | 0.109 |
| journal cost | -0.091 | 0.149 |
