# Supplementary material for: Why we publish where we do: Faculty publishing values and their relationship to review, promotion and tenure expectations
Source: PLoS One. 2020 Mar 11;15(3):e0228914. doi: 10.1371/journal.pone.0228914 (PMC7065820; doi:10.1371/journal.pone.0228914)
Supplement: S4 Table — (DOCX) [file pone.0228914.s004.docx]

| S4 Table. Spearman's correlations and p values for perception of the RPT process by age | | |
| --- | --- | --- |
| **Variable** | **Age** | **p value** |
| rpt blog | 0.160 | 0.010 |
| rpt book chapter | 0.122 | 0.045 |
| rpt book | -0.011 | 0.852 |
| rpt pub numbers | -0.026 | 0.664 |
| rpt performance | 0.317 | 0.000 |
| rpt media | -0.046 | 0.459 |
| rpt pre print | -0.037 | 0.581 |
| rpt open access | 0.250 | 0.000 |
| rpt society | 0.104 | 0.092 |
| rpt journal IF | -0.080 | 0.189 |
| rpt journal name | -0.065 | 0.281 |
| rpt pub total | -0.058 | 0.332 |
