# Supplementary material for: Why we publish where we do: Faculty publishing values and their relationship to review, promotion and tenure expectations
Source: PLoS One. 2020 Mar 11;15(3):e0228914. doi: 10.1371/journal.pone.0228914 (PMC7065820; doi:10.1371/journal.pone.0228914)
Supplement: S5 Table — ANOVA were used for statistical significance tests. (DOCX) [file pone.0228914.s005.docx]

| S5 Table. Mean responses and p values for perception of the RPT process by tenure status. ANOVA were used for statistical significance tests. | | | | | |
| --- | --- | --- | --- | --- | --- |
|  | **Tenured** | **SE** | **Non-tenure** | **SE** | **p=** |
| rpt blog | 1.86 | 1.10 | 1.74 | 0.95 | 0.227 |
| rpt book chapter | 3.61 | 1.39 | 3.14 | 1.26 | 0.009 |
| rpt book | 4.34 | 1.56 | 3.74 | 1.58 | 0.025 |
| rpt pub numbers | 5.32 | 0.94 | 5.22 | 1.15 | 0.391 |
| rpt performance | 2.20 | 1.57 | 2.19 | 1.57 | 0.978 |
| rpt media | 3.07 | 1.51 | 3.03 | 1.50 | 0.597 |
| rpt pre print | 2.30 | 1.35 | 2.18 | 1.34 | 0.552 |
| rpt open access | 2.21 | 1.40 | 1.88 | 1.21 | 0.072 |
| rpt society | 3.56 | 1.55 | 3.21 | 1.53 | 0.075 |
| rpt journal IF | 4.59 | 1.37 | 4.85 | 1.37 | 0.521 |
| rpt journal name | 3.84 | 1.21 | 4.82 | 1.33 | 0.600 |
| rpt pub total | 3.36 | 0.99 | 5.51 | 0.88 | 0.419 |
