# Supplementary material for: Why we publish where we do: Faculty publishing values and their relationship to review, promotion and tenure expectations
Source: PLoS One. 2020 Mar 11;15(3):e0228914. doi: 10.1371/journal.pone.0228914 (PMC7065820; doi:10.1371/journal.pone.0228914)
Supplement: S6 Table — ANOVA were used for statistical significance tests. (DOCX) [file pone.0228914.s006.docx]

| S6 Table. Mean responses and p values for perception of the RPT process by gender and institution type. ANOVA were used for statistical significance tests. | | | | | | | | | | | |
| --- | --- | --- | --- | --- | --- | --- | --- | --- | --- | --- | --- |
| **Variable** | **Female** | **SE** | **Male** | **SE** | **p=** | **R Type** | **SE** | **M Type** | **SE** | **p=** |  |
| rpt blog | 1.89 | 1.12 | 1.76 | 1.00 | 0.322 | 1.79 | 1.03 | 1.90 | 1.14 | 0.480 |  |
| rpt book chapter | 3.63 | 1.36 | 3.35 | 1.38 | 0.106 | 3.29 | 1.30 | 3.86 | 1.42 | 0.001 |  |
| rpt book | 4.35 | 1.49 | 3.98 | 1.66 | 0.060 | 4.07 | 1.58 | 4.38 | 1.59 | 0.139 |  |
| rpt pub numbers | 5.51 | 0.88 | 5.11 | 1.05 | 0.001 | 5.35 | 0.98 | 5.19 | 1.03 | 0.220 |  |
| rpt performance | 2.22 | 1.50 | 2.19 | 1.63 | 0.931 | 2.17 | 1.51 | 2.24 | 1.65 | 0.773 |  |
| rpt media | 3.11 | 1.53 | 3.04 | 1.49 | 0.746 | 3.02 | 1.45 | 3.04 | 1.56 | 0.939 |  |
| rpt pre print | 2.29 | 1.38 | 2.26 | 1.33 | 0.852 | 2.14 | 1.28 | 2.64 | 1.47 | 0.043 |  |
| rpt open access | 2.06 | 1.39 | 2.17 | 1.33 | 0.525 | 2.01 | 1.19 | 2.30 | 1.58 | 0.102 |  |
| rpt society | 3.58 | 1.66 | 3.34 | 1.44 | 0.214 | 3.49 | 1.46 | 3.46 | 1.73 | 0.892 |  |
| rpt journal IF | 4.82 | 1.38 | 4.50 | 1.35 | 0.057 | 4.81 | 1.25 | 4.37 | 1.54 | 0.014 |  |
| rpt journal name | 4.92 | 1.24 | 4.75 | 1.25 | 0.265 | 4.97 | 1.12 | 4.58 | 1.39 | 0.013 |  |
| rpt pub total | 5.60 | 0.82 | 5.20 | 1.05 | 0.001 | 5.45 | 0.88 | 5.30 | 1.11 | 0.212 |  |
