# Supplementary material for: Why we publish where we do: Faculty publishing values and their relationship to review, promotion and tenure expectations
Source: PLoS One. 2020 Mar 11;15(3):e0228914. doi: 10.1371/journal.pone.0228914 (PMC7065820; doi:10.1371/journal.pone.0228914)
Supplement: S7 Table — Total n = 154. (DOCX) [file pone.0228914.s007.docx]

| S7 Table. Ordered logistic model predicting merit pay as a factor in publishing decisions (Model 1). Total n= 154. | | | | | | |
| --- | --- | --- | --- | --- | --- | --- |
| **Variable** | **Odds Ratio** | **Std. Err** | **z** | **P value** | **95% confidence interval** | |
| age | 1.268 | 0.221 | 1.36 | 0.174 | 0.901 | 1.785 |
| gender | 0.812 | 0.290 | -0.58 | 0.559 | 0.403 | 1.635 |
| r-type | 1.671 | 0.659 | 1.30 | 0.193 | 0.771 | 3.620 |
| tenured | 0.590 | 0.259 | -1.20 | 0.230 | 0.249 | 1.396 |
| pubs published | 1.067 | 0.202 | 0.34 | 0.730 | 0.737 | 1.545 |
| rpt pub numbers | 0.657 | 0.154 | -1.79 | 0.073 | 0.414 | 1.040 |
| rpt preprint | 1.083 | 0.156 | 0.55 | 0.581 | 0.816 | 1.436 |
| rpt open access | 1.355 | 0.190 | 2.16 | 0.031 | 1.028 | 1.784 |
| rpt society | 1.045 | 0.124 | 0.37 | 0.711 | 0.828 | 1.319 |
| rpt journal IF | 1.039 | 0.163 | 0.24 | 0.807 | 0.764 | 1.414 |
| rpt journal name | 0.928 | 0.157 | -0.44 | 0.659 | 0.666 | 1.293 |
| rpt pub total | 1.305 | 0.322 | 1.08 | 0.281 | 0.805 | 2.117 |
