# Supplementary material for: Why we publish where we do: Faculty publishing values and their relationship to review, promotion and tenure expectations
Source: PLoS One. 2020 Mar 11;15(3):e0228914. doi: 10.1371/journal.pone.0228914 (PMC7065820; doi:10.1371/journal.pone.0228914)
Supplement: S8 Table — Total n = 203. (DOCX) [file pone.0228914.s008.docx]

| S8 Table. Ordered logistic model predicting readership respondents want to reach as a factor in publishing decisions (Model 2). Total n= 203. | | | | | | |
| --- | --- | --- | --- | --- | --- | --- |
| **Variable** | **Odds Ratio** | **Std Err** | **z** | **P value** | **95% confidence interval** | |
| age | 0.934 | 0.139 | -0.46 | 0.648 | 0.697 | 1.252 |
| gender | 1.135 | 0.339 | 0.43 | 0.671 | 0.632 | 2.039 |
| r-type | 0.905 | 0.292 | -0.31 | 0.757 | 0.480 | 1.705 |
| tenured | 1.273 | 0.460 | 0.67 | 0.503 | 0.628 | 2.583 |
| pubs published | 1.679 | 0.291 | 2.99 | 0.003 | 1.196 | 2.359 |
| rpt pub numbers | 1.104 | 0.189 | 0.58 | 0.561 | 0.790 | 1.544 |
| rpt preprint | 1.096 | 0.127 | 0.79 | 0.430 | 0.873 | 1.375 |
| rpt open access | 1.365 | 0.168 | 2.54 | 0.011 | 1.073 | 1.737 |
| rpt society | 0.956 | 0.088 | -0.49 | 0.623 | 0.798 | 1.144 |
| rpt journal IF | 0.806 | 0.104 | -1.68 | 0.094 | 0.627 | 1.037 |
| rpt journal name | 1.252 | 0.176 | 1.60 | 0.110 | 0.950 | 1.650 |
| rpt pub total | 1.318 | 0.232 | 1.57 | 0.117 | 0.933 | 1.861 |
