# Supplementary material for: Why we publish where we do: Faculty publishing values and their relationship to review, promotion and tenure expectations
Source: PLoS One. 2020 Mar 11;15(3):e0228914. doi: 10.1371/journal.pone.0228914 (PMC7065820; doi:10.1371/journal.pone.0228914)
Supplement: S9 Table — Total n = 203. (DOCX) [file pone.0228914.s009.docx]

| S9 Table. Ordered logistic model predicting Journal Impact Factor as a factor in publishing decisions (Model 3). Total n= 203. | | | | | | |
| --- | --- | --- | --- | --- | --- | --- |
| **Variable** | **Odds Ratio** | **Std Err** | **z** | **P value** | **95% confidence interval** | |
| age | 0.911 | 0.124 | -0.69 | 0.492 | 0.697 | 1.190 |
| gender | 0.905 | 0.250 | -0.36 | 0.717 | 0.527 | 1.555 |
| r-type | 0.853 | 0.256 | -0.53 | 0.596 | 0.474 | 1.536 |
| tenured | 0.605 | 0.214 | -1.42 | 0.155 | 0.302 | 1.209 |
| pubs published | 1.320 | 0.204 | 1.80 | 0.072 | 0.976 | 1.787 |
| rpt pub numbers | 1.066 | 0.172 | 0.39 | 0.693 | 0.777 | 1.462 |
| rpt preprint | 1.246 | 0.137 | 2.00 | 0.045 | 1.005 | 1.545 |
| rpt open access | 0.986 | 0.109 | -0.12 | 0.902 | 0.794 | 1.226 |
| rpt society | 0.969 | 0.084 | -0.36 | 0.716 | 0.818 | 1.148 |
| rpt journal IF | 1.884 | 0.249 | 4.79 | 0.000 | 1.454 | 2.442 |
| rpt journal name | 0.939 | 0.132 | -0.45 | 0.653 | 0.713 | 1.236 |
| rpt pub total | 0.907 | 0.153 | -0.58 | 0.565 | 0.652 | 1.263 |
