# Supplementary material for: Why we publish where we do: Faculty publishing values and their relationship to review, promotion and tenure expectations
Source: PLoS One. 2020 Mar 11;15(3):e0228914. doi: 10.1371/journal.pone.0228914 (PMC7065820; doi:10.1371/journal.pone.0228914)
Supplement: S10 Table — Total n = 204. (DOCX) [file pone.0228914.s010.docx]

| S10 Table. Ordered logistic model predicting society journals as a factor in publishing decisions (Model 4). Total n= 204. | | | | | | |
| --- | --- | --- | --- | --- | --- | --- |
| **Variable** | **Odds Ratio** | **Std Err** | **z** | **P value** | **95% confidence interval** | |
| age | 1.373 | 0.186 | 2.34 | 0.019 | 1.053 | 1.789 |
| gender | 1.026 | 0.277 | 0.10 | 0.924 | 0.604 | 1.743 |
| r-type | 1.277 | 0.379 | 0.82 | 0.410 | 0.714 | 2.285 |
| tenured | 0.698 | 0.235 | -1.07 | 0.286 | 0.361 | 1.351 |
| pubs published | 1.127 | 0.166 | 0.81 | 0.419 | 0.844 | 1.504 |
| rpt pub numbers | 0.795 | 0.138 | -1.32 | 0.187 | 0.566 | 1.118 |
| rpt preprint | 0.995 | 0.101 | -0.05 | 0.962 | 0.816 | 1.213 |
| rpt open access | 1.065 | 0.115 | 0.58 | 0.561 | 0.861 | 1.317 |
| rpt society | 1.720 | 0.160 | 5.83 | 0.000 | 1.433 | 2.064 |
| rpt journal IF | 0.914 | 0.113 | -0.73 | 0.465 | 0.718 | 1.164 |
| rpt journal name | 1.101 | 0.153 | 0.69 | 0.491 | 0.838 | 1.446 |
| rpt pub total | 1.167 | 0.192 | 0.94 | 0.348 | 0.845 | 1.613 |
