# Supplementary material for: Why we publish where we do: Faculty publishing values and their relationship to review, promotion and tenure expectations
Source: PLoS One. 2020 Mar 11;15(3):e0228914. doi: 10.1371/journal.pone.0228914 (PMC7065820; doi:10.1371/journal.pone.0228914)
Supplement: S11 Table — Total n = 205. (DOCX) [file pone.0228914.s011.docx]

| S11 Table. Ordered logistic model predicting journal/venue/publisher they regularly read as a factor in publishing decisions (Model 5). Total n= 205. | | | | | | |
| --- | --- | --- | --- | --- | --- | --- |
| **Variable** | **Odds Ratio** | **Std Err** | **z** | **P value** | **95% confidence interval** | |
| age | 1.057 | 0.147 | 0.40 | 0.688 | 0.806 | 1.388 |
| gender | 0.825 | 0.227 | -0.70 | 0.486 | 0.481 | 1.417 |
| r-type | 1.207 | 0.363 | 0.63 | 0.531 | 0.670 | 2.175 |
| tenured | 0.767 | 0.258 | -0.79 | 0.430 | 0.397 | 1.482 |
| pubs published | 1.248 | 0.182 | 1.52 | 0.128 | 0.938 | 1.661 |
| rpt pub numbers | 1.009 | 0.163 | 0.05 | 0.957 | 0.734 | 1.386 |
| rpt preprint | 1.072 | 0.114 | 0.65 | 0.515 | 0.870 | 1.320 |
| rpt open access | 1.063 | 0.116 | 0.56 | 0.576 | 0.858 | 1.317 |
| rpt society | 1.052 | 0.091 | 0.59 | 0.554 | 0.889 | 1.246 |
| rpt journal IF | 0.843 | 0.097 | -1.49 | 0.135 | 0.673 | 1.055 |
| rpt journal name | 1.309 | 0.165 | 2.15 | 0.032 | 1.024 | 1.675 |
| rpt pub total | 0.980 | 0.155 | -0.13 | 0.900 | 0.719 | 1.337 |
