# Supplementary material for: Why we publish where we do: Faculty publishing values and their relationship to review, promotion and tenure expectations
Source: PLoS One. 2020 Mar 11;15(3):e0228914. doi: 10.1371/journal.pone.0228914 (PMC7065820; doi:10.1371/journal.pone.0228914)
Supplement: S12 Table — Total n = 203. (DOCX) [file pone.0228914.s012.docx]

| S12 Table. Ordered logistic model predicting journal/venue/publisher their peers regularly read as a factor in publishing decisions (Model 6). Total n= 203. | | | | | | |
| --- | --- | --- | --- | --- | --- | --- |
| **Variable** | **Odds Ratio** | **Std Err** | **z** | **P value** | **95% confidence interval** | |
| age | 0.876 | 0.122 | -0.95 | 0.341 | 0.667 | 1.151 |
| gender | 1.314 | 0.368 | 0.98 | 0.329 | 0.759 | 2.276 |
| r-type | 0.971 | 0.288 | -0.10 | 0.922 | 0.543 | 1.738 |
| tenured | 0.753 | 0.262 | -0.82 | 0.414 | 0.381 | 1.488 |
| pubs published | 0.982 | 0.148 | -0.12 | 0.901 | 0.731 | 1.319 |
| rpt pub numbers | 1.001 | 0.162 | 0.01 | 0.994 | 0.729 | 1.374 |
| rpt preprint | 0.884 | 0.094 | -1.15 | 0.248 | 0.718 | 1.090 |
| rpt open access | 1.114 | 0.123 | 0.98 | 0.326 | 0.898 | 1.382 |
| rpt society | 1.055 | 0.092 | 0.61 | 0.543 | 0.888 | 1.253 |
| rpt journal IF | 0.891 | 0.107 | -0.96 | 0.336 | 0.703 | 1.128 |
| rpt journal name | 1.376 | 0.185 | 2.38 | 0.018 | 1.058 | 1.791 |
| rpt pub total | 1.146 | 0.183 | 0.85 | 0.393 | 0.838 | 1.568 |
