# Supplementary material for: Why we publish where we do: Faculty publishing values and their relationship to review, promotion and tenure expectations
Source: PLoS One. 2020 Mar 11;15(3):e0228914. doi: 10.1371/journal.pone.0228914 (PMC7065820; doi:10.1371/journal.pone.0228914)
Supplement: S13 Table — Total n = 203. (DOCX) [file pone.0228914.s013.docx]

| S13 Table. Ordered logistic model predicting how often a journal is cited as a factor in publishing decisions (Model 7). Total n= 203. | | | | | | |
| --- | --- | --- | --- | --- | --- | --- |
| **Variable** | **Odds Ratio** | **Std Err** | **z** | **P value** | **95% confidence interval** | |
| age | 0.829 | 0.113 | -1.37 | 0.170 | 0.634 | 1.084 |
| gender | 1.069 | 0.285 | 0.25 | 0.804 | 0.634 | 1.802 |
| r-type | 0.747 | 0.219 | -0.99 | 0.320 | 0.421 | 1.327 |
| tenured | 1.148 | 0.399 | 0.40 | 0.691 | 0.581 | 2.269 |
| pubs published | 1.107 | 0.161 | 0.70 | 0.485 | 0.832 | 1.473 |
| rpt pub numbers | 0.794 | 0.135 | -1.36 | 0.175 | 0.569 | 1.108 |
| rpt preprint | 1.258 | 0.134 | 2.15 | 0.031 | 1.021 | 1.551 |
| rpt open access | 0.930 | 0.099 | -0.68 | 0.494 | 0.755 | 1.145 |
| rpt society | 0.892 | 0.076 | -1.33 | 0.183 | 0.754 | 1.055 |
| rpt journal IF | 1.572 | 0.196 | 3.63 | 0.000 | 1.231 | 2.008 |
| rpt journal name | 1.053 | 0.141 | 0.38 | 0.703 | 0.809 | 1.369 |
| rpt pub total | 1.374 | 0.232 | 1.89 | 0.059 | 0.988 | 1.912 |
