# Supplementary material for: Why we publish where we do: Faculty publishing values and their relationship to review, promotion and tenure expectations
Source: PLoS One. 2020 Mar 11;15(3):e0228914. doi: 10.1371/journal.pone.0228914 (PMC7065820; doi:10.1371/journal.pone.0228914)
Supplement: S14 Table — Total n = 205. (DOCX) [file pone.0228914.s014.docx]

| S14 Table. Ordered logistic model predicting overall prestige of the journal/venue/publisher as a factor in publishing decisions (Model 8). Total n= 205. | | | | | | |
| --- | --- | --- | --- | --- | --- | --- |
| **Variable** | **Odds Ratio** | **Std Err** | **z** | **P value** | **95% confidence interval** | |
| age | 0.895 | 0.124 | -0.80 | 0.424 | 0.682 | 1.175 |
| gender | 1.310 | 0.369 | 0.96 | 0.338 | 0.754 | 2.275 |
| r-type | 0.736 | 0.220 | -1.02 | 0.305 | 0.409 | 1.323 |
| tenured | 0.592 | 0.209 | -1.48 | 0.138 | 0.297 | 1.183 |
| pubs published | 1.093 | 0.162 | 0.60 | 0.549 | 0.817 | 1.463 |
| rpt pub numbers | 1.112 | 0.190 | 0.62 | 0.534 | 0.796 | 1.554 |
| rpt preprint | 1.180 | 0.126 | 1.55 | 0.122 | 0.957 | 1.455 |
| rpt open access | 0.827 | 0.095 | -1.64 | 0.100 | 0.660 | 1.037 |
| rpt society | 1.057 | 0.091 | 0.64 | 0.524 | 0.892 | 1.252 |
| rpt journal IF | 1.174 | 0.142 | 1.33 | 0.182 | 0.927 | 1.487 |
| rpt journal name | 1.420 | 0.189 | 2.64 | 0.008 | 1.095 | 1.843 |
| rpt pub total | 0.909 | 0.154 | -0.57 | 0.572 | 0.652 | 1.266 |
