# Supplementary material for: Why we publish where we do: Faculty publishing values and their relationship to review, promotion and tenure expectations
Source: PLoS One. 2020 Mar 11;15(3):e0228914. doi: 10.1371/journal.pone.0228914 (PMC7065820; doi:10.1371/journal.pone.0228914)
Supplement: S15 Table — Total n = 202. (DOCX) [file pone.0228914.s015.docx]

| S15 Table. Ordered logistic model predicting public availability of the publication (i.e. open access) as a factor in publishing decisions (Model 9). Total n= 202. | | | | | | |
| --- | --- | --- | --- | --- | --- | --- |
| **Variable** | **Odds Ratio** | **Std Err** | **z** | **P value** | **95% confidence interval** | |
| age | 1.103 | 0.151 | 0.72 | 0.473 | 0.843 | 1.443 |
| gender | 1.333 | 0.367 | 1.04 | 0.296 | 0.777 | 2.287 |
| r-type | 0.840 | 0.258 | -0.57 | 0.569 | 0.460 | 1.532 |
| tenured | 0.658 | 0.222 | -1.24 | 0.215 | 0.339 | 1.275 |
| pubs published | 1.115 | 0.166 | 0.73 | 0.467 | 0.832 | 1.493 |
| rpt pub numbers | 1.038 | 0.170 | 0.23 | 0.820 | 0.753 | 1.431 |
| rpt preprint | 1.033 | 0.107 | 0.31 | 0.754 | 0.843 | 1.266 |
| rpt open access | 1.957 | 0.225 | 5.84 | 0.000 | 1.562 | 2.452 |
| rpt society | 0.885 | 0.082 | -1.32 | 0.185 | 0.738 | 1.061 |
| rpt journal IF | 0.981 | 0.114 | -0.17 | 0.869 | 0.782 | 1.231 |
| rpt journal name | 1.129 | 0.157 | 0.87 | 0.383 | 0.860 | 1.482 |
| rpt pub total | 1.006 | 0.164 | 0.04 | 0.971 | 0.730 | 1.386 |
