# Supplementary material for: Why we publish where we do: Faculty publishing values and their relationship to review, promotion and tenure expectations
Source: PLoS One. 2020 Mar 11;15(3):e0228914. doi: 10.1371/journal.pone.0228914 (PMC7065820; doi:10.1371/journal.pone.0228914)
Supplement: S16 Table — Total n = 192. (DOCX) [file pone.0228914.s016.docx]

| S16 Table. Ordered logistic model predicting journal cost to publish as a factor in publishing decisions (Model 10). Total n= 192. | | | | | | |
| --- | --- | --- | --- | --- | --- | --- |
| **Variable** | **Odds Ratio** | **Std Err** | **z** | **P value** | **95% confidence interval** | |
| age | 0.866 | 0.124 | -1.01 | 0.315 | 0.653 | 1.147 |
| gender | 0.396 | 0.114 | -3.21 | 0.001 | 0.225 | 0.698 |
| r-type | 1.261 | 0.394 | 0.74 | 0.458 | 0.684 | 2.325 |
| tenured | 1.514 | 0.530 | 1.18 | 0.236 | 0.762 | 3.006 |
| pubs published | 1.159 | 0.174 | 0.99 | 0.324 | 0.864 | 1.555 |
| rpt pub numbers | 1.169 | 0.204 | 0.90 | 0.370 | 0.831 | 1.646 |
| rpt preprint | 1.113 | 0.119 | 1.00 | 0.317 | 0.903 | 1.372 |
| rpt open access | 1.041 | 0.114 | 0.37 | 0.713 | 0.840 | 1.290 |
| rpt society | 0.926 | 0.085 | -0.84 | 0.400 | 0.773 | 1.108 |
| rpt journal IF | 1.070 | 0.131 | 0.55 | 0.580 | 0.841 | 1.362 |
| rpt journal name | 0.850 | 0.115 | -1.20 | 0.231 | 0.652 | 1.109 |
| rpt pub total | 0.956 | 0.165 | -0.26 | 0.794 | 0.681 | 1.341 |
