# Supplementary material for: Why we publish where we do: Faculty publishing values and their relationship to review, promotion and tenure expectations
Source: PLoS One. 2020 Mar 11;15(3):e0228914. doi: 10.1371/journal.pone.0228914 (PMC7065820; doi:10.1371/journal.pone.0228914)
Supplement: Attachment — Submitted filename: Response to Reviewer.docx [file pone.0228914.s017.docx]

Dear Editor and Reviewers,

Thank you and the three reviewers for the helpful feedback on our manuscript “Why we publish where we do: Faculty publishing values and their relationship to review, promotion and tenure expectations”. We are pleased to resubmit our manuscript with the requested revisions. Below you will find the reviewers’ feedback (in gray) interspersed with a description of how we addressed each of the points raised (in black).

We would like to take this opportunity to express our appreciation to the reviewers for you’re their thoughtful feedback. We are convinced that we have adequately addressed all of the expressed concerns and that the manuscript has been improved as a result of this process.

Sincerely,

Juan Pablo Alperin

Assistant Professor, Publishing

Associate Director, Public Knowledge Project

Director, Scholarly Communications Lab

Simon Fraser University

Reviewer #1: This article provides interesting data on faculty perceptions of publishing. This is a valuable and timely area of study. However, the paper would need to undergo significant revisions in order to meet the PLOS ONE publishing criteria (namely: (3) Experiments, statistics, and other analyses are performed to a high technical standard and are described in sufficient detail; and (4) Conclusions are presented in an appropriate fashion and are supported by the data.) I describe these concerns as well as other comments on improving the manuscript below.

The introduction assumes a North American context for academe, without explicitly framing it as such. It specifically references the growth of English-language journals and describes scenarios that apply predominately to North American universities. By taking for granted this context, it frames North American as the normative context for academic structure. I would strongly encourage the authors to reframe the introduction to acknowledge this context explicitly, rather than implicitly.

Response: Thank you for this helpful feedback. We have added into the first sentence this context about the setting being particularly relevant for high-income Western countries.

The last three research questions acknowledge that this is perception-based. The first question should also acknowledge that these are self-reported factors; the actual influence of these issues is difficult to measure post-hoc and via self-report. The authors should include a limitation section that acknowledges that what authors report as their rationale for choosing a publication may be an idealized version of reality. This may be another explanation for the “mismatch” observed.

Response: We have changed the first question to reflect a perceptions basis similar to the other four questions. In addition we have added a limitations section to the end of the discussion to mark this limitation as well as the geographic focus.

What exactly is meant by “pseudo-randomly”? Why manual selection rather than the assignment of a number to each faculty member and the use of a random number generator? This description of the sampling procedure reduces confidence.

Furthermore, and perhaps more importantly, it is unclear why the sampling used the sample frame of institutions derived from the previous study (Alperin et al., 2019). Given that no analysis was done linking responses with policies, there is no justification for this sample, which may not have been the most appropriate frame for the research questions posed. More justification for this choice is necessary.

Response: While we agree that doing a fully random selection would be preferred, the manual collection of faculty names and email addresses made it impossible to do so. In some cases, departments had less than 5 faculty, so that all were selected. In others, faculty lists were split across multiple pages, so we attempted to move across them and pick faculty from each page. We removed reference to the term pseudo-randomly and opted for the simpler language found inside the parenthesis.

It is true that, in the present study, no analysis was done linking to the policy documents. However, this sample was part of a larger project in which the relationship is studied. We have now articulated this. While the sample was not chosen for the specific purpose of answering these research questions, in the following paragraph (p. 3) we explain the breakdown of Canadian vs US institutions, and across the various institution types.

The authors need to use caution in the interpretation and reporting of Likert-type questions. Means should not be used for not ratio data. For example, the authors examine the number of publications per year, in binned categories. They then conclude that women publish fewer articles than men—citing that their mean is 3.05 compared to 3.28, where a score of 3 indicates 1-2 publications and 4 indicates 3-5 publications. The authors should only use modal amounts and categorical analyses such as Chi-squared for these data.

Response: We have rerun the statistical tests for pubspublished, the categorical variable that we include in our analysis. We did this for the relationship of sex and r-type/m-type institution to pubpublished, using a chi-square test. We have updated the manuscript and supplementary materials with the new data.

In Table 2, it is essential to note that two things are skewing this: the first is that one-third of the respondents are not from research-intensive universities, yet they may consider peers to be at all universities. Therefore, it may not be necessarily incorrect that things like funding and JIF have higher importance to their peers.

Response: The reviewer is making a hypothesis on why we observe differences between faculty’s reported values and their perceptions of their peer’s values. We don’t believe that it would be appropriate to make such a hypothesis in the results section, especially since we do not have data that speaks to who faculty consider to be their peers. Making assumptions about who faculty at B- or M-type institutions are measuring themselves against is likely to be contested, and so our approach has been to plainly report the data.

More to the point, who people consider to be their “peers” does not affect our findings or conclusions. We also do not believe there is a “correct” or “incorrect” way for faculty to think about themselves in relation to others. What is important, we believe, is that people see themselves as different than others on some dimensions.

(As a small note of concern, the authors round 32.4% up to 33% in the description of the respondents; this is non-standard and should be fixed.)

Response: This was a copyediting mistake on our part. We have changed the 33% to 32%.

Furthermore, the majority of respondents are past-tenure; therefore, it again might be true that these things matter less. This may be less of a gap between perceptions of self and perceptions of others, but rather the fact that the selves and the peers are not equally represented in these data.

Response: Similar to the above, we think that what is important is that faculty perceive themselves as different from their peers—whoever they consider their peers to be. The reviewer is essentially suggesting that we should be comparing faculty’s values to others who are “similar” to themselves, but there is no clear way on determining who is “similar” beyond doing what we have done, which is to let faculty self-identify those they consider to be “peers.”

Finally—and this is not a minor point—when we looked at what faculty think is important in the RTP process (Table 3), neither the type of institution nor the tenure status came up as significant factors.

I do not find convincing the statement that there is a “mismatch between faculty’s own values and how they perceive those of their peers”. The second paragraph of the discussion seems rather to support the interpretation that this is a mismatch of institutional culture and academic status. The entire discussion, however, seems to focus on this disconnect, which does not appear to be substantiated. The logic of the discussion seems highly problematic in that it at once acknowledges a difference in expectations by career stage (which is well-documented) and then suggests that junior faculty are somehow living under a false reality because they have different expectations and values than their tenured peers.

Response: We respectfully disagree that our discussion of this “mismatch” is not substantiated. For example, in Table 2 we demonstrate, through statistical tests, that faculty clearly perceive themselves to care more about open access and readership than their peers, where their peers they perceive to care more about prestige oriented factors like impact factor or being paid for publications in specific journals. Furthermore, we do not present evidence that pre-tenured faculty have different expectations or values than their tenured peers- Table 2 is all of the respondents together, not just pre-tenure. We do find that older and tenured faculty are more likely to value things such as books in the RPT process and untenured faculty are more likely to value the prestige oriented components of publishing; however, this is not the “mismatch” that we highlight in the discussion. Instead, as noted earlier, the mismatch is between what faculty themselves (regardless of their career stage) on average value versus what they on average think their peers value. The other mismatch we refer to in the discussion is related to values faculty consider in their own publishing decisions versus those that faculty think matter for RPT. Here we find that there is an mismatch in individual’s perceptions of themselves and in what they themselves consider valuable for RPT, rather than a mismatch between their perception of self versus others. In this sense, we highlight the example of open access, which faculty say they themselves value (and to a greater degree than their peers, see Table 2) but that the same faculty overwhelmingly report is not an important consideration in RPT. In this sense, there is a potential mismatch of values versus reality: faculty value open access, but may not be able to realize it because they don’t think it will “count” in the RPT process.

The authors fail to cite the most relevant and contemporary research in the introduction and discussion. For example, in the discussion of gendered differences in the responses, they fail to note the considerable research on funding/resources (e.g., Ginther, Duch), attrition, or the productivity puzzle. The majority of their references are more than a decade old. In fact, they only cite four journal articles other than their own published in the last decade. For another topic, this would be irrelevant; however, given that they are making claims about the state of contemporary research and that more recent articles are available, I would encourage them to conduct an appropriate review of the literature. This will allow them to better motivate the article, to contextualize it in previous knowledge, and to demonstrate the contribution of this work.

Response: Thank you for the suggested additional references. We have included additional references on this topic in our discussion (including now five additional papers, all published 2012 or later).

Related to this, the authors state that the work builds upon Alperin (2019), but this is not listed in the references. Given that the reader is referred to this for an explanation of the methods, this is a major concern. (The work cannot be evaluated without the information in this reference and it is not available in the manuscript.)

Response: This was an omission on our part, which we have now corrected.
